# Complete mitochondrial genome and phylogenetic analysis of *Ixodes ovatus* (Acari: Ixodidae)

**DOI:** 10.1080/23802359.2022.2122745

**Published:** 2022-11-11

**Authors:** Dandan Jiang, Xinyan Lu, Chunhong Du, Shanbin Hu, Zhipang Huang, Xing Yang

**Affiliations:** a School of Public Health, Dali University, Dali, P. R. China; b Integrated Laboratory of Pathogenic Biology, College of Preclinical Medicine, Dali University, Dali, P. R. China; c Yunnan Institute of Endemic Diseases Control and Prevention, Yunnan, P. R. China; d Adminstration of Yunling Provincial Nature Reserve, Nujiang, Yunnan, PR China; e Institute of Eastern-Himalaya Biodiversity Research, Dali University, Yunnan, China

**Keywords:** *Ixodes ovatus*, Complete mitogenome, Phylogenetic analysis

## Abstract

*Ixodes ovatus* is referred to as an obligatory blood-sucking ectoparasite that is capable to infest both humans and animals. In the present study, the complete mitochondrial genome of *I. ovatus* was sequenced and analyzed using next-generation sequencing (NGS) technology. With a size of 14,520 bp, the entire mitogenome contains 37 genes including 13 protein-coding genes (PCGs), 22 transfer RNAs (tRNAs), 2 ribosomal RNAs (rRNAs), and 3 control regions (D-loops). Based on the 13 PCGs nucleotide sequences, the phylogenetic relationship of *I. ovatus* was analyzed using Maximum-likelihood. As suggested by the results of the obtained phylogenety, *I. ovatus* is most closely associated with *Ixodes hexagonus.* This study is expected to promote further studies on the evolution of Ixodidae.

*Ixodes ovatus* (Neumann, 1899) is a species of hard tick with a wide geographical distribution across China, Taiwan, Korea, Japan, Burma, and Thailand (Harry Hoogstraal 1973). With cows, horses, donkeys, deer, and sheep as known hosts, *I. ovatus* can accidentally parasitize humans, thus spreading a wide variety of severe animal and human pathogens, including *Anaplasma phagocytophilum*, *Borrelia miyamotoi*, *Borrelia garinii*, and *Babesia spp.*, etc. (Shimada 2003). Therefore, correct identification of tick species is essential for disease control (Shimada 2003). However, conventional morphological identification requires extensive experience, which may restrict its applications. Since the mitochondrial genome is regarded as an effective genetic marker for species identification, sequencing of their complete mitochondrial genomes is beneficial to the identification and classification of ticks (Taanman 1999).

In this study, *I. ovatus* samples were collected in March 2021 from Tue Village, Nujiang City, Yunnan Province, China (98°48′59.84″E, 26°34′20.56″N) and then preserved in 75% ethanol. The recognition of samples was conducted by Professor Chunhong Du based on the morphological characteristics (Lu et al. 2021). Then, the specimen was deposited in Parasitological Museum, Dali University NO. DLUP2103 (URL: http://www.dali.edu.cn/jcyxy/xkpt/jcyxsyjxzx/6431.htm, Contact person: Xing Yang, yang08220013@163.com). The entire DNA was extracted using the standard CTAB technique, for storage at −20 °C before use (Lu et al. 2021). The mitochondrial genome was sequenced on the Illumina NovaSeq platform (Shanghai Personal Biotechnology Co, Ltd) which was assembled using A5-miseq software (Coil et al. 2015), and genome annotations were performed using the Swiss-Prot web server (http://www.ebi.ac.uk/uniprot/).

The mitochondrial genome of *I. ovatus* was determined as 14,520 bp (Genbank accession no. OM317739), involving 13 PCGs (*cytb, nad1-6, atp8, nad4L, cox1-3,* and *atp6*), 22 tRNAs, 2 rRNAs. The genetic order of the *I. ovatus* was identical to hard ticks. The entire base composition of the *I. ovatus* mitochondrial genome was determined as 37.54% A, 37.39% T, 16.24% C, 8.83% G. The size of *I. ovatus* small submit rRNA and large submit rRNA was 657 and 1,190 bp, respectively. It was discovered that the length of 22 tRNAs varied from 55 bp (*tRNA-Ser*) to 71 bp (*tRNA-Lys*), with 13 tRNAs encoded on the plus-strand (Thomas et al. 2013).

The GTR + G + I model was applied as the suitable model for sequencing, and the maximum-likelihood method was adopted through the MEGA 7.0 software with 1000 bootstrap replicates (Kumar et al. 2016). As shown in Figure 1, the phylogenetic tree included complete mitogenomes sequences of 28 Ixodidae species previously published on GenBank. As revealed by the phylogenetic analysis, the obtained tree is divided into two phylogroups: Metastriata and Prostriata (Ciloglu et al. 2021). Additionally, it is shown that *I. ovatus* and all the other species within the genus *Ixodes* cluster in a branch with high statistical support, confirming *I. ovatus* within the genus *Ixodes.* The complete mitochondrial genome of *I. ovatus* provides an important molecular resource for further study on the phylogeny of the genus *Ixodes* and of Ixodidae (Kelava et al. 2021).

**Figure 1.**
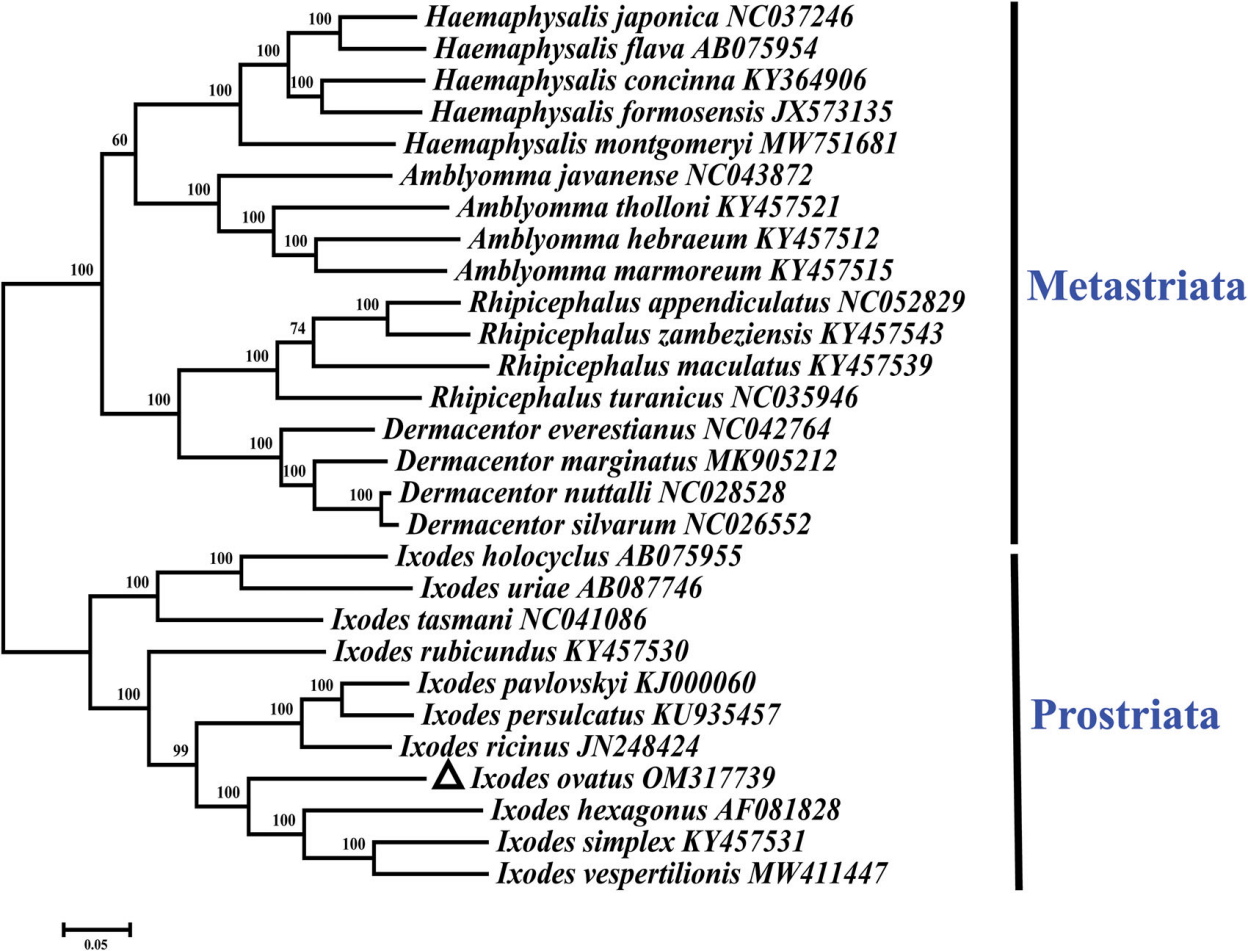
Maximum-likelihood (ML) phylogeny of 28 species of the family Ixodidae based on the 13 concatenated nucleotide sequences of protein-coding genes (PCGs), utilizing the GTR + G + I model and after 1,000 bootstrap replications. The black triangle sign represents the species in this study. Bootstrap support values are shown above the nodes.

## Ethical approval

This study was approved by the Administration Committee of Experimental Animals, Dali University, Yunnan Province, China.

## Data Availability

The data that support the findings of this study are openly available in the National Center for Biotechnology Information (NCBI) at https://www.ncbi.nlm.nih.gov. The accession number of the complete mitochondrial genome is OM317739. The associated BioProject, SRA, and Bio-Sample numbers are PRJNA820144, SRR18494365 and SAMN26982089, respectively.
